# A Doctor Discovers Michelangelo’s Self-Portrait in San Bartholomew’s Flayed Skin in the Sistine Chapel

**DOI:** 10.25122/jml-2018-0001

**Published:** 2018

Dr. Francesco La Cava, a physician who wrote a lot about the history of medicine, had a strong passion for art that allowed us to see a particular anatomical detail. Dr. La Cava, looking at the Universal Judgment in the Sistine Chapel, believed he could see the face of Michelangelo.

It was well known that Michelangelo did not like painting portraits and it seemed that he had never painted one, but through his observations, the doctor realized what no one had ever noticed before; Michelangelo’s face was inscribed in the skin, still fresh and bleeding, of Saint Bartholomew. The image he saw resembled the known portraits of the great painter. We can also try to reflect as the doctor did. In the Universal Judgment, one can see Bartholomew holding a knife in his right hand, on display to the Redeemer, and in his other hand, he holds his flayed skin, the evidence of his martyrdom. Michelangelo’s anatomical and physiological knowledge can be seen in the precise representation of body details and the tension of the limbs of the saint. This consideration can also be observed in his definition of skin, which he is clutching at a posterior point, closer to the left side than the right; the skin was folded and held tight to reinforce the seal, similar to the methods used by anatomist experts who performed autopsies. The occipital skin point from which the skin of the head is hanging shows that it is inanimate, fresh in scarring and therefore yielding. The head is in a natural position, the vertical axis forming a top-to-bottom line, while the face falls to the right. The head falls to the front around the occiput, its point of attachment, so the lower part of the face, mouth, and chin, sink between the skin folds of the chest. The right limbs are lower than the left ones, the right forearm being in a pronounced state. La Cava was convinced it was a self-portrait of Michelangelo: the face of Bartholomew is badly painted, while the skin shows abundant hair; there are differences in the shape of the nose, in the eyes and other anatomic details. We do not know the mysterious intention that prompted Michelangelo to portray his face in the flayed skin, but we realize that Michelangelo was a master not only in depicting perfect bodies but also in painting a flayed skin, perhaps with the experience of having observed them for real.

**Figure:**
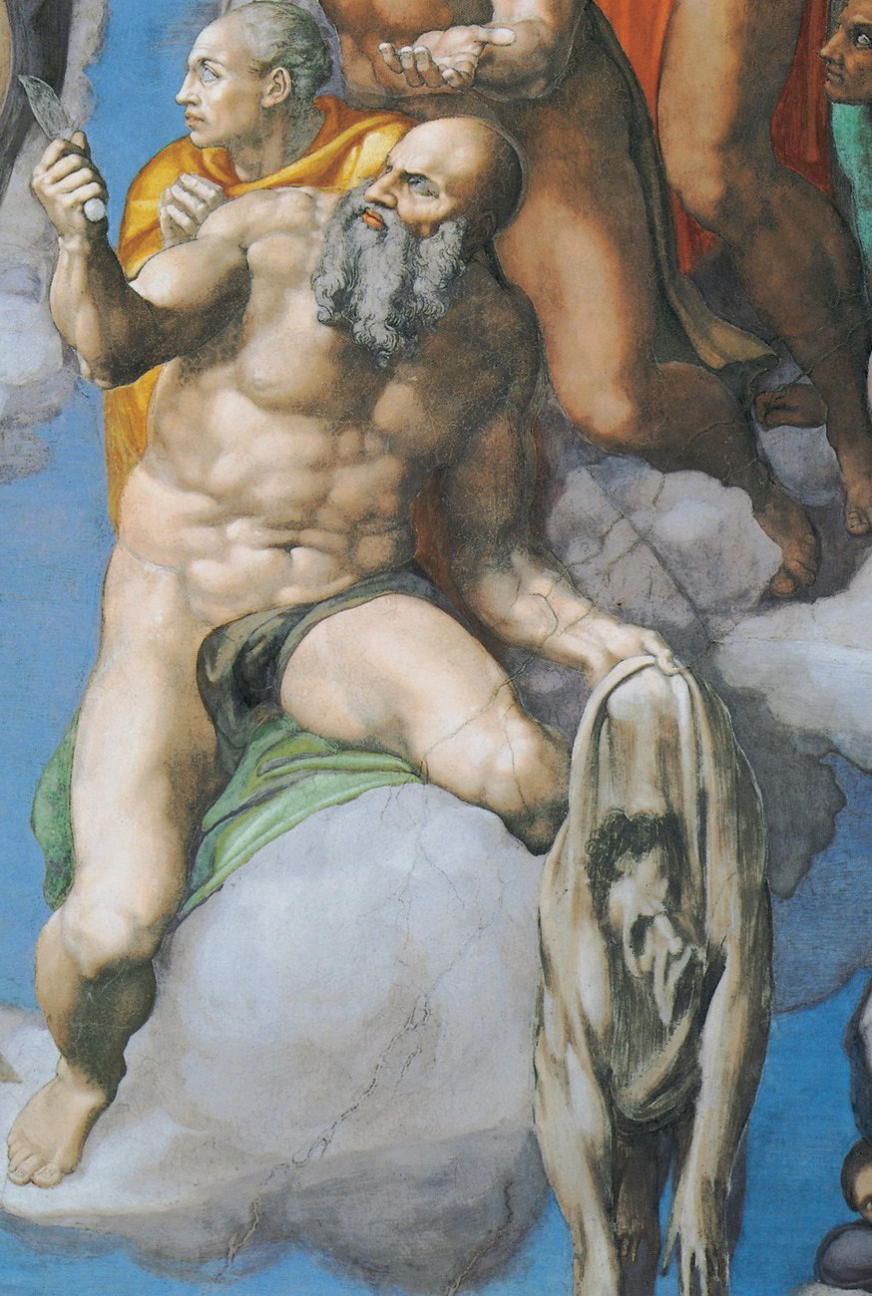


## Conflict of Interest

The authors confirm that there are no conflicts of interest.
