# Correction to: A Doctor Discovers Michelangelo’s Self-Portrait in San Bartholomew’s Flayed Skin in the Sistine Chapel

**Published:** 2018

**Authors:** Marta Licata

**Affiliations:** Centre of Research of Osteoarchaeology and Paleopathology, Department of Biotechnology and Life Sciences, University of Insubria, Varese, Italy

## Abstract

In the original publication, the author name and the corresponding author field have been omitted. The author name is: Marta Licata. The original article is now corrected.

In the original publication J Med Life 2018; 11:257-8[1], the author name and the corresponding author field have been omitted. The author name is: Marta Licata. The original article is now corrected. We wish to apologise for any misunderstanding or inconvenience caused.
